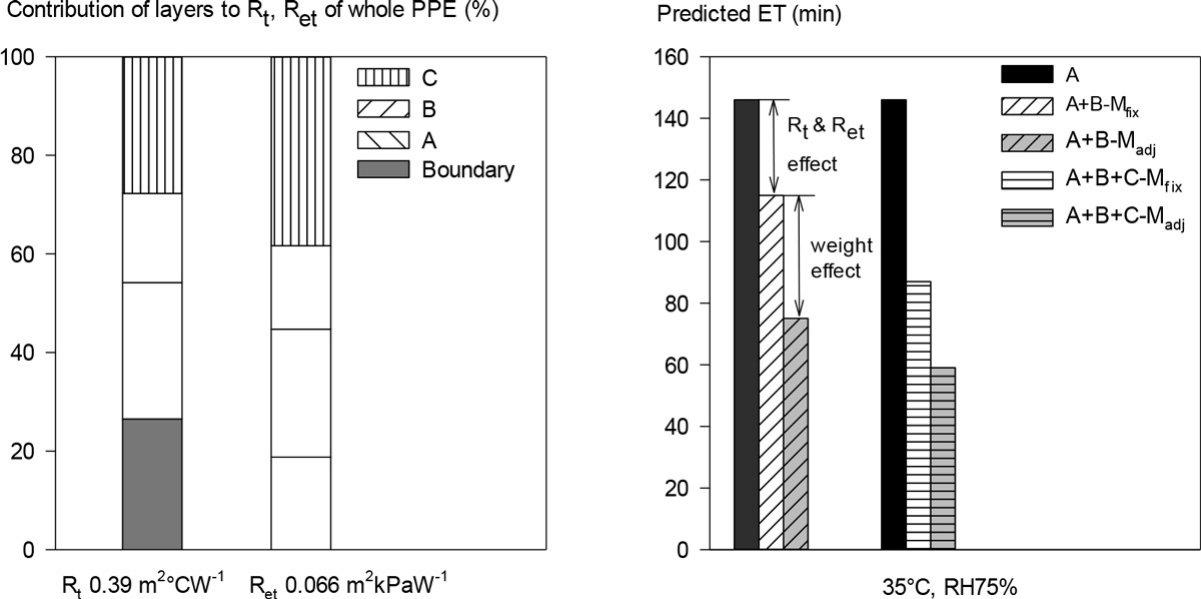# Quantitative evaluation of personal protective ensembles relative to heat strain

**DOI:** 10.1186/2046-7648-4-S1-A133

**Published:** 2015-09-14

**Authors:** Xiaojiang Xu, Julio Gonzalez

**Affiliations:** 1 US Army Research Institute of Environmental Medicine Biophysics/Biomedical Modeling Division, Natick, MA 01760, USA

## Introduction

Personal protective equipment (PPE) exacerbates heat strain experienced by users through: (a) increases in thermal (R_t_) and evaporative (R_et_) resistances; and (b) increases in metabolic rate ($\overset{\cdot }{\text{M}}$) during physical activity driven in large part by ensemble weight. This study aimed to quantify the effects of PPE R_t _& R_et _and ensemble weight on heat strain during walking.

## Methods

Stepwise thermal manikin (TM) testing and modeling were used to analyse a three-layer PPE ensemble (weight 37.4 kg). Layers: uniform (A); body armour and combat load (B); chemical protective clothing (C). The PPE was tested on a TM to measure R_t _& R_et_, starting with layer A and then adding an additional layer in each step. $\overset{\cdot }{\text{M}}$ during walking at 1.22 m.s^-1^, adjusted (${\overset{\cdot }{\text{M}}}_{\text{adj}}$) for the layer weight, were 300, 404 and 428W for configurations with A, A+B and A+B+C, respectively. A human thermoregulatory model was used to predict endurance time (ET, min) for each configuration at a fixed $\overset{\cdot }{\text{M}}$ (${\overset{\cdot }{\text{M}}}_{\text{fix}}$) of 300 W and at its ${\overset{\cdot }{\text{M}}}_{\text{adj}}$. ET was defined as time needed for the core temperature to rise to 39 °C.

## Results

The left figure indicates the fractional contribution of each layer to R_t _& R_et _of the whole system (A+B+C). The right figure is the predicted ET, showing influences of B or B+C in comparison with A. The difference between A and A+B-${\overset{\cdot }{\text{M}}}_{\text{fix}}$ indicates ET reduction due to R_t _& R_et _with added B, and the difference between A+B-${\overset{\cdot }{\text{M}}}_{\text{fix}}$ and A+B-${\overset{\cdot }{\text{M}}}_{\text{adj}}$ indicates ET reduction due to the weight of B. Thus compared with ET for A of 146 min, the R_t _& R_et _of B reduce ET by 31 min while the added weight reduces ET by 40 min further. Similarly, the increased R_t _& R_et _of B+C reduce ET by 59 min, while the added weight reduces ET by 28 min.

## Discussion

This study (a) reveals the fractional contributions of PPE resistances by layer, (b) demonstrates the effects of PPE weight on ET and quantifies ET reduction due to increases in $\overset{\cdot }{\text{M}}$ associated with PPE weights, and (c) isolate the contributions of two different PPE properties, R_t _& R_et _and ensemble weight, to predicted heat strain. Impacts of each PPE layer on ET can be quantified by this approach.

## Conclusion

This study provides a new systematic approach to understanding more the aetiology of heat strain, and to designing PPE to maximise user protection while minimizing heat strain.

**Figure 1:**